# Who Reads Food Labels? Selected Predictors of Consumer Interest in Front-of-Package and Back-of-Package Labels during and after the Purchase

**DOI:** 10.3390/nu12092605

**Published:** 2020-08-27

**Authors:** Paweł Bryła

**Affiliations:** Department of International Marketing and Retailing, Faculty of International and Political Studies, University of Lodz, Narutowicza 59a, 90-131 Lodz, Poland; pawel.bryla@uni.lodz.pl; Tel.: +48-426655830

**Keywords:** food label use, front-of-package (FOP) labels, back-of-package (BOP) labels, nutrition claims, health claims, nutrition knowledge, consumer behavior

## Abstract

The paper aims to identify selected predictors of food label use to extend our knowledge about consumer behavior related to food purchases. Two types of information were examined: front-of-package (FOP) and back-of-package (BOP), and two contexts of reading labels were distinguished: during shopping and at home. Various types of potential predictors were tested, including demographic (e.g., age, gender, household size, place of living), socioeconomic (e.g., education, professional activity, income), behavioral (e.g., purchasing certain types of products), and psychographic (e.g., importance attached to various types of information) criteria. The survey was conducted with the use of the CAWI (Computer-Assisted Web Interviews) methodology in a sample of 1051 Polish consumers. Quota sampling was applied based on sex, age, education, place of living (urban vs. rural), and region. Descriptive statistics, *t*-tests, ANOVAs, Pearson correlation coefficients, and multiple and retrograde step regressions were applied. In retrograde step regression models, only one predictor (self-rated knowledge about nutrition healthiness) turned out to be significant for all four measures of label reading. The remaining predictors were specific to selected measures of reading labels. The importance of the information about the content of fat and that about the health effects of consuming a food product were significant predictors of three types of food label use. This study confirms the necessity to investigate reading labels in fine-grained models, adapted to different types of labels and different contexts of reading. Our results show that demographic or socioeconomic variables are not significant predictors of reading food labels for a large group of Polish consumers.

## 1. Introduction

In previous research, food label use was associated with consumer characteristics, product type, and purchasing context. The following variables were found to affect reading labels: gender [1,2,3,4,5], age [4,6], marital status [7], ethnicity [2], socioeconomic status [2,7], including education level [4,5,7,8,9], professional activity [9], income [5], place of living—rural or urban areas [2,5], Body Mass Index [2,4], being an athlete [10], food-related motivation [11], nutrition knowledge [4,12,13], self-reported health [4,10], having a special diet [4,9], being concerned with a healthy lifestyle [10], attitude towards the health value of the products [13], health orientation [14], taste [9,13], price [9], product specificity [12], buying the product for the first time [12], the amount of time spent shopping [9], and buying organic food [15]. There are various connections between these predictors, e.g., the gender effect is mainly due to differences in nutrition knowledge levels [12]. Health-related variables were the most important group of predictors of food label use, followed by motivating factors and sociodemographic variables. Placing importance on health, healthy eating, and nutritional value of food, perceived vulnerability for diet-related diseases, nutrition knowledge, numeracy, and gender were positively associated with the frequency of food label use [16]. Subjective norms and diet-health concern were significant predictors of intention to use food labels [17]. Self-efficacy and health literacy were predictors of food label use among young adults [18]. Consumers’ level of nutrition knowledge influenced their ability to process food labels. Experts used the central route processing to scrutinize intrinsic cues and make judgments about food products, while novices used the peripheral route processing to make simple inferences about the extrinsic cues in labels [19]. Nutritional knowledge and attitude toward food label use positively predicted food label use through self-efficacy and trust. However, these mediation effects were moderated by gender such that the indirect relationship was stronger among men than women [20]. In their review [21], Soederberg Miller and Cassady found an overreliance on convenience samples relying on younger adults, limiting our understanding of how knowledge supports food label use in later life. This limitation is overcome in the current study, as it is based on a sample representative by age.

It is also important to distinguish between food label use at the point of purchase and at home. Education has a positive impact on the likelihood of using labels at home but not while shopping. The average amount of time spent on grocery shopping per visit affects label use while shopping but not at home. The same applies to the primary source of nutrition information being books, magazines, radio, TV, or newspapers. Moreover, being on a special diet influenced label reading in the shop but not at home. The importance of price while shopping affected reading labels at home only, and the importance of taste was a significant predictor of reading labels while shopping only [9]. These results support the need to study food label use separately while shopping and at home.

Findings consistently support a relationship between label reading and dietary practices [22,23]. Changes in diet quality due to label use were estimated for different types of label information. Consumer label use increased the average Healthy Eating Index. These improvements differed across label information types that were used. Among nutritional panels, serving sizes, nutrient content claims, a list of ingredients, and health claims, the use of health claims on food labels provided the highest level of improvement in diet quality [24]. Search for total fat, saturated fat, and cholesterol information on food labels was less likely among individuals who consumed more of the three nutrients, respectively. The search was also related to perceived benefits and costs of using the label, the perceived capability of using the label, knowledge of nutrition and fats, perceived efficacy of diets in reducing the risk of illnesses, perceived importance of nutrition in food shopping, perceived importance of a healthy diet, and awareness of a linkage between excessive consumption of the nutrients and health problems [25]. Nutrition knowledge had a strong effect on general label use, degree of use, and on use of nutrient content concerning fat, ingredients, and vitamins/minerals [26]. Subjects with chronic diseases were more aware of nutritional recommendations, checked more often for specific nutrients, and used nutrition information on food labels more often than did participants without such diseases. However, label use behavior was inconsistently associated with dietary guideline compliance [27]. The odds of healthy weight loss behaviors were two to four times higher when food labels were used frequently to seek information on calories and nutrients such as total fat, saturated fat, or cholesterol [28]. A recent review [29], by Anastasiou, Miller, and Dickinson, demonstrated that results were inconsistent in reporting a relationship between diet and food label use but indicated that reading the nutrition facts label was associated with healthier diets, measured by food frequency questionnaires and 24 h recalls. However, there is insufficient research on the association between dietary consumption and the use of ingredients lists, serving size information, and front-of-package (FOP) labels. Nutrition label use decreased the risk of poor dietary quality regardless of poverty status [30]. General food label use was the main determinant of diet quality and partly mediated the association between eating behavior traits and diet quality. The stronger mediating effect observed in men suggested they relied more on food labeling when attempting to restrain themselves, which translated into better diet quality [31].

It is also important to recognize that various food label types have different effects on consumer behavior. Compared with individual characteristics, nutrition label types had an increased impact on food product ranking ability [32]. Different food label formats differed in the understanding of consumers. The multiple traffic light (MTL) labels influenced the perceived healthiness of foods most often [33]. In an international, large-scale survey, viewing the MTL provided the most favorable ratings out of five FOP nutrition label formats (health star rating, MTL, Nutri-Score, reference intakes, and warning label) [34]. The perceived usefulness and public support of mandatory implementation were higher for the MTL than for the Nutri-Score label [35]. Nutri-Score, which is a summary, interpretive, polychromatic FOP label, emerged as the most effective in the Bulgarian context [36]. FOP nutrition labels were more likely to be viewed than Nutrition Facts labels [37]. Different degrees of health-orientation were reflected in the diverse use of labeled information. Highly health-oriented consumers were more likely to refer to the extensive information reported on the nutrition facts panel, whereas claims were of main interest for consumers with a low orientation to health [14]. Furthermore, 52.5% of consumers did not read the ingredients’ list written on the food label [38]. Simplified FOP labels can induce healthier purchases compared with when only back-of-package (BOP) nutrition information is available [39]. Clearer BOP labeling is also needed [40]. Nutritional warnings cause a salience bias that makes excessive nutrient content and its negative health consequences more salient in consumers’ minds, especially in the case of products with a particular health-related connotation [41]. Although FOP labels may help shoppers make healthier food choices, those advocating for effective labels must resist opposition from food corporations [42]. Future opportunities for FOP labeling include the potential for integrating nutritional profiles with non-nutrient factors affecting health such as food processing and environmental sustainability [43]. Although FOP labels help consumers to identify healthier products, their ability to nudge consumers toward healthier choices is more limited. Importantly, FOP labels may lead to halo effects, positively influencing not only virtue but also vice products, e.g., interpretive nutrient-specific labels improve health perceptions of both vice and virtue products, yet they influence only the purchase intention of virtues [44].

The paper aims to identify selected predictors of reading food labels with the distinction of the label type (FOP and BOP), and the context of food label use (during shopping and afterward, at home). Therefore, four measures of reading labels were considered in this study: FOP in the shop, BOP in the shop, FOP at home, and BOP at home. The main contribution of this paper lies in systematically analyzing predictors of reading labels from the perspective of two major label types (FOP and BOP) and two main reading contexts (in the shop and at home) based on a survey in a large, representative, nation-wide sample of consumers.

## 2. Materials and Methods

The survey was conducted with the use of the CAWI (Computer Assisted Web Interviews) methodology. The execution of the survey was commissioned to a specialized market research agency. The respondents were informed that the results would be used only for scientific purposes, respecting the anonymity principle. The sample size amounted to 1051 consumers. The target sample size was set at a comparable level with previous consumer studies conducted at the national level in Poland [45,46,47]. It was aimed to obtain a sample resembling the general population of Polish adults, regarding four criteria: age, sex, education level, and the size of the city of origin (in particular, the urban/rural divide). Quota sampling was applied based on five criteria: sex (men and women), age (the following age intervals: 15–24, 25–34, 35–44, 45–54, 55–64, 65 and more), education (primary, secondary and tertiary), place of living (urban and rural areas), and region (all 16 Polish regions). Thanks to this approach, the sample structure was similar to the general population of Polish consumers according to the above-mentioned criteria (Table A1).

The questionnaire was created by the author of this study. It was composed of elements adapted from previous research using validated tools [14,48,49,50,51,52,53,54,55,56,57,58,59]. It consisted of 38 questions. The full questionnaire is included in a book in Polish [60]. The operationalization of the key variables used in this study is provided in Table A2. Reading labels was investigated in 4 configurations: (1) FOP in the point of purchase (FOP shop), (2) BOP in the point of purchase (BOP shop), (3) FOP after purchase (FOP home), (4) BOP after purchase (BOP home). It was operationalized as a percentage share of food products the respondent buys. Some more difficult terms were explained in the questionnaire, in particular health claims, nutrition claims, and quality signs. The research examined general food labels. My dependent variables referred to FOP and BOP labels without specifying which information was put on which label. However, in some questions, I examined the importance attached to particular types of information placed on the labels, such as health claims, nutrition claims, list of ingredients, expiry date, country of origin, cooking recipes, brand, organic certificate, quality signs, recommendations of scientific institutes, price, as well as selected types of nutritional information (energy value, the content of fat, sugar, salt, protein, vitamins, dietary fiber, Omega-3 fatty acids) and health information (lowering cholesterol, reducing the risk of heart diseases, strengthening bones, impact on the digestive system, reducing tiredness and fatigue, maintaining proper vision, proper development of children, and proper functioning of the heart). Various types of potential predictors were tested, including demographic (e.g., age, gender, household size, place of living), socioeconomic (e.g., education, professional activity, income), behavioral (e.g., purchasing certain types of products), and psychographic (e.g., importance attached to various types of information) criteria. For most of the questions, 5-point Likert scales were used.

First, I tested the differences in reading food labels depending on selected demographic and socioeconomic criteria: sex, age, place of living, education, professional activity, income, household size, and the number of children. Second, I checked whether reading labels differed depending on some purchasing habits: buying dietary supplements, organic food, functional food, and fair trade products. Third, I examined label use according to selected criteria related to one’s health and diet. Fourth, I examined the correlations of food label use with the evaluation of the quantity, understandability, and credibility of selected information on the food packaging.

Next, I tested the associations between food label use and the importance attached to selected types of information placed on the labels. Four questions were devoted to this issue. The first one concerned general food labels. Second, I focused on the importance of information at the first purchase of a given product. This question referred to a product that was new to the consumer and not necessarily to the entire market Third, I took into consideration typical types of nutritional information. Last, I examined the correlations with selected types of health-related information.

Furthermore, the associations between food label use and the importance attached to marketing communication instruments were identified. The following marketing communication options were put forward for the respondents’ evaluation: TV commercials, producer website, retailer website, producer social media profile, retailer social media profile, consumer opinions in social media, mobile applications, recommendations of family or friends, recommendations of a dietician, outdoor advertising (e.g., billboards), advertising newsletters of retailers, articles in press and magazines, TV culinary programs, culinary blogs, product packaging, and shop assistant.

Regarding information used in the marketing communication for food products, the following options were considered: health effects of consuming a given product, care for the natural environment, supporting producers (e.g., farmers), low price, national origin of the product, the utility of the product in a certain diet, above-average quality of the product, and traditional method of production.

Based on statistical differences revealed in *t*-tests and ANOVAs as well as the most significant Pearson correlations, 32 independent variables were selected to be analyzed in multiple regressions. Separate regression models were constructed for each type of reading labels.

In the data analysis, descriptive statistics, *t*-tests, analyses of variance (ANOVAs), Pearson correlation coefficients, and multiple and retrograde step regressions were applied. T-tests were used to compare two quantitative results, ANOVAs were used to compare multiple quantitative results, Pearson correlation coefficients were used to examine the linear correlations between quantitative variables, the multiple regression models were used to examine the simultaneous influence of all independent variables separately on the 4 types of reading labels, and the retrograde step regressions were used to narrow down the set of independent variables to those that remained significant at the ***p* < 0.05** level. Independent variables that differentiated any measure of reading labels in a statistically significant way were included in separate multiple regression models for FOP shop, BOP shop, FOP home, and BOP home. To obtain more parsimonious models, only those predictors that reached statistical significance at the level of ***p* < 0.05** were accepted in retrograde step regressions. The analyses were conducted in Statistica 12 (TIBCO Software Inc., Palo Alto, CA, USA). I report continuous *p*-values instead of thresholds, following the recent guidelines of statisticians [61]. Results that are statistically significant at ***p* < 0.05** are boldfaced to increase their visibility.

## 3. Results

Women read food labels more often than men, but the sex differences reached statistical significance only for reading BOP labels at the point of purchase (BOP shop) and FOP labels after the purchase at home (FOP home) (Table 1).

A series of ANOVAs demonstrated that age (measured in 10-year intervals) did not differentiate any measure of reading labels (Table 2).

Reading labels was not affected by the place of living (defined as rural areas, towns up to 50,000, cities of 50–500,000, and cities of more than 500,000) either (Table 3).

As far as the education level is concerned, only the FOP shop was influenced in a significant way (Table 4). Respondents who had tertiary education read food labels in shops more often than the other education groups.

As far as the professional activity is concerned, it affected reading labels at the point of purchase—both FOP and BOP—but not after the purchase at home. White-collar workers read food labels most frequently in-store (both FOP and BOP) (Table 5).

Reading food labels was associated with household income only for the FOP shop. It was highest among respondents living in households with middle income (3001–4000 PLN) and the highest income (over 6000 PLN per month) (Table 6).

Only FOP label reading (in the shop and at home) correlated positively and significantly with the size of one’s household (Table 7).

Reading food labels was associated with purchasing certain types of products, namely dietary supplements, organic food, functional food, and fair trade products (Table 8). Buying dietary supplements increased the frequency of reading labels on the front of the package in the shop and at home as well as on the back of the package at home only. Buying organic food affected all measures of reading labels in a highly significant way. Buying functional food influenced all kinds of reading labels except for the FOP shop. Buying fair trade products differentiated significantly reading only the BOP information.

Reading food labels was correlated with self-rated healthiness of one’s diet, one’s knowledge about healthy nutrition, and self-rated health but not BMI (Body Mass Index) (Table 9).

However, none of the investigated measures of reading labels depended on being on a special diet for health reasons (Table 10).

Reading BOP labels was related to the evaluation of the quantity of selected information (health claims, nutrition claims, and quality signs) on food labels (Table 11). Those who considered that there was too little such information tended to read food labels more often. Moreover, all measures of reading labels were associated with the self-evaluated understandability of information put on the labels (namely health claims, nutrition claims, quality signs, functional food, and organic food). Finally, reading labels correlated with the perceived credibility of such information (health claims, nutrition claims, list of ingredients, expiry date, organic certificate, and quality signs). Those who trusted information placed on the labels more tended to read them more often both in the shop and at home.

Reading food labels depended on the importance attached to selected information put on them (Table 12). The subjective importance of almost all types of information correlated positively and significantly with reading FOP and BOP food labels both at the point of purchase and at home, except cooking recipes, the importance of which was correlated significantly only with the FOP home.

As far as the importance of information at the first purchase of a food product (excluding price) is concerned, it differentiated only BOP label reading, both in the shop and at home. The respondents indicating the list of ingredients read the highest share of BOP labels in both contexts (Table 13).

All the investigated nutritional information types correlated significantly with all four measures of reading labels (Table 14).

All of the investigated health information types correlated significantly with all the measures of reading labels (Table 15).

Furthermore, reading food labels was associated with the importance attached to all investigated promotion instruments for food products with health and nutrition claims, with some minor exceptions for particular food label use measures (Table 16).

All kinds of reading labels were associated the most strongly with the importance attached to the health effects of consuming a given product (Table 17).

It is also worth noting that all the analyzed types of reading labels correlated significantly with the willingness to pay (WTP) a higher price for food products with health claims and nutrition claims compared to conventional products (Table 18). The strongest correlation was observed between the BOP shop and the WTP for nutrition claims.

The multiple regression models explained better reading BOP labels (BOP shop R^2^ = 0.173, BOP home R^2^ = 0.143) than FOP labels (FOP shop R^2^ = 0.091, FOP home R^2^ = 0.089) (Table 19). In the full multiple regression model, reading FOP labels at the point of purchase was determined by only three predictors: self-rated knowledge about healthy nutrition, the importance of the expiry date information on the food packaging, and the importance of the information about the health effects of consuming a given product. Second, reading BOP information in the shop turned out to depend on five variables. It was positively associated with the importance attached to the list of ingredients, self-rated nutrition knowledge, indicating the list of ingredients as the most important information at the first purchase, and average assessment of the quantity of selected information on the food packaging: health claims, nutrition claims, and quality signs. It was negatively related to the consumer education level. The third regression model reported in this table refers to reading FOP labels at home. Six variables had a significant impact on this measure, four out of which were positively related: self-rated nutrition knowledge, the importance attached to culinary blogs, the importance of information about the health effects of consuming the product, and the importance of the information that the product reduces tiredness and fatigue. On the other hand, it was negatively associated with the information about the impact of the product on the digestive system and the importance attached to the recommendations of a dietician. The last regression model concerned the predictors of reading BOP information after the purchase. It was found to depend on five variables, all of which contributed positively: the importance of the information about the content of fat, the importance of the list of ingredients, the importance of the information about the health effects of consuming a product, the evaluation of the quantity of information on the label, and the importance of the list of ingredients when one buys a food product for the first time.

According to the final retrograde step regression models (Table 20), the FOP shop increased with being a white-collar worker, having better (self-rated) knowledge about healthy nutrition, evaluating the credibility of information on labels higher, attaching importance to the expiry date, and health effects in the marketing communication for food products. Second, the BOP shop increased with one’s knowledge about a healthy diet, better self-rated health, evaluating the quantity of information on labels as insufficient, attaching importance to the list of ingredients in general and during first-time purchases, and attaching importance to the content of fat. Third, reading FOP labels at home increased with the knowledge about a healthy diet, attaching importance to the content of fat and to the impact of the product on lowering cholesterol, attaching importance to culinary blogs, and to health effects in the marketing communications but decreased with the importance attached to the recommendations of a dietician. Finally, reading BOP labels at home depended on buying functional food, one’s knowledge about a healthy diet, evaluation of the quantity of information on food labels, the importance attached to the list of ingredients, to the content of fat, and to health effects in the marketing communication.

## 4. Discussion

The main contribution of this paper is the identification of variables that influence food label use in four configurations: FOP in the shop, BOP in the shop, FOP at home, and BOP at home, based on a survey in a large, representative, nation-wide sample of consumers. In retrograde step regression models, only one predictor (self-rated knowledge about healthy diet) turned out to be significant for all four measures of label reading. Two more predictors (importance attached to the content of fat and health effects in marketing communication) were significant in three out of the four investigated forms of reading labels. The remaining independent variables were specific to one or two forms only. It confirms the necessity to study reading labels in fine-grained models, adapted to different types of labels and different contexts of reading. Moreover, this study demonstrated that sociodemographic and behavioral characteristics have limited power of explaining reading labels in multivariate models (except being a white-collar worker for FOP shop and buying functional food for BOP home). Most of the relevant predictors were psychographic, as they concerned the importance attached to selected information types put on the labels or used in the marketing communication as well as the evaluation of the quantity and credibility of information placed on the labels. It is also worth noting that most of the predictors were related to the nutrition and health aspects of communication.

My results differed from some previous findings. In a sample of US adults, significant differences in food label use were observed across all demographic characteristics examined [62]. This difference may stem from: (1) international differences in food label use, (2) a different operationalization of food label use as well as (3) the inclusion of a wide set of psychographic criteria in my multivariate models, which turned out to be better predictors. In another study, determinants of food label use differed by sex. Age and diet quality perception were significant predictors of food label use for both men and women, but ethnicity was significant for males only. Similar to my findings, women checked food label components more often than men [63], but the current study distinguishes various forms and contexts of food label use. Significant differences between men and women were observed for reading BOP labels in the shop and FOP labels at home. Reading food labels has different predictors than the importance attached to some types of information placed on the labels. For instance, another study based on the same dataset demonstrated that the importance attached to salt content information depended on sex and age [64]. Following most previous studies, my results confirmed the impact of nutrition knowledge on food label use. However, interestingly, this relationship may be treated as bidirectional. Cavaliere et al. found that food label use increased the nutritional knowledge of consumers, which in turn favored a healthy diet [65]. It is also important to realize that the valuation of information on the labels is heterogeneous across consumer segments. For instance, Ballco and De Magistris [66] identified three consumer segments: “health-claims oriented”, “nutritional- and health-claim oriented”, and “indifferent”. There is a complexity of targeting nutrition labels because a nonlinear effect of health attitude on the selection of products with increased nutrients content was revealed in previous research [67].

Information may not always be effective in improving food choices. One explanation is that nutrition information is complex and difficult to convey in a clear, actionable manner. In addition, knowledge, while necessary, may not be sufficient to motivate behavior change [68]. The online shopping environment offers new promising tools, such as dynamic food labels with real-time feedback [69], which are not available offline. Even though food label use was associated with improved dietary factors, it was not sufficient alone to modify behavior ultimately leading to improved health outcomes [62]. The perceived credibility of nutrition claims, agreeing that the availability of health-related information is not sufficient for the vast majority of consumers to change their food preferences, and believing that foods carry an excessive number of nutrition claims affected the importance of nutrition claims among food processors and distributors [70]. There is also evidence of mistrust in health claims, as indicated by the negative relationship between the consideration of such claims and the stated importance of “quality” and perceived need to “change dietary quality”—the more discerning shoppers were the least likely to consider health claims [71].

There are a few limitations to this study. The first one is that it is based on self-reported data only. A known limitation of self-report instruments such as surveys and questionnaires is their susceptibility to socially desirable responding. Socially desirable responding is the tendency to give answers that make the respondent look good, or the tendency ‘‘to stretch the truth in an effort to make a good impression’’ [72]. In my survey, I minimized this bias by ensuring the survey anonymity and confidentiality of answers. At the beginning of the questionnaire, the respondents were informed that the survey was anonymous and the results would be used for scientific purposes only. Both self-reported and objective measures of food label use were positively associated with dietary quality. However, self-reported measures appeared to capture a greater motivational component of food label use than did more objective measures [73]. Second, the determination coefficients in my regression models were relatively low, especially for reading FOP labels. This means that there may be other important predictors, which were not included in my study. Nevertheless, taking into consideration the wide range of potential predictors that were examined in my models, it may be also due to the difficulty of assessing the percentage of products the labels of which are read by the given respondent. I opted for the percentage measure rather than a Likert-type scale of frequency because of its higher objectivity. Responses such as “often” or “seldom” can be understood by different consumers differently, e.g., for some people reading 30% of labels may mean “often” and for others “seldom”. That is why I preferred the percentage measures, bearing in mind also the weakness of this measure related to the different numerical skills of respondents. This shortcoming of my study may be overcome by applying a combination of observational methods (ethnography) and surveys in future research.

There are several implications of my results. First, nutrition knowledge improvement programs should be developed, as higher nutrition knowledge was found to translate into a higher interest in reading all kinds of food labels (FOP and BOP) in both contexts (during the purchase and after the purchase), and food label use leads to more favorable food choices for public health. Second, food processors and retailers should more often consider the possibility of emphasizing the health effects of a given product alongside or even instead of other attributes. Third, consumers are particularly sensitive to information about the content of fat. Therefore, the use of nutrition claims and graphical designs of the packaging pointing to the low-fat or zero-fat properties of a product is encouraged. Fourth, retailers should encourage their customers to read the labels at the point of purchase, which may be achieved by allocating special space and offering tools to obtain additional information about the products. Fifth, smartphone apps should be developed to facilitate consumer understanding of information placed on the labels and to facilitate comparisons across products in a given category. Sixth, it is important to pay attention not only to the type of information placed on the labels but also its quantity, and last but not least, credibility. The manufacturers should be selective and focus on the consumer-friendly design of labels. Public authorities should facilitate the use of official nutrition claims and health claims, at the same time keeping high standards of awarding such claims. Public information campaigns should be organized to explain the transparency of the procedures of obtaining health and nutrition claims and the credibility of other types of information on the labels. Finally, public authorities should increase their effort in monitoring the industry so that it adheres to high standards of labeling, including the accuracy, completeness, and visibility of information sought by consumers.

## Figures and Tables

**Table 1:** Reading food labels by sex.

Reading Labels (%)	Women	Men	t	*p*
FOP shop	56.72	53.77	1.726	0.085
BOP shop	52.59	47.69	2.888	**0.004**
FOP home	51.49	46.29	2.763	**0.006**
BOP home	52.86	50.04	1.502	0.133

**Table 2:** Reading food labels by age.

Reading Labels (%)	Age Intervals						ANOVA	
	15–24	25–34	35–44	45–54	55–64	≥65	F	*p*
FOP shop	59.75	55.15	53.17	53.87	54.50	55.81	1.160	0.327
BOP shop	51.94	50.57	50.34	47.75	50.70	50.24	0.372	0.868
FOP home	48.80	49.17	47.55	47.26	51.84	49.57	0.479	0.792
BOP home	53.63	51.99	51.38	48.10	53.68	50.46	0.746	0.589

**Table 3:** Reading food labels by the place of living.

Reading Labels (%)	Place of Living				ANOVA	
	Rural Areas	<50,000	50,000–500,000	>500,000	F	*p*
FOP shop	54.20	58.34	55.30	54.66	1.016	0.385
BOP shop	48.89	51.78	51.16	50.45	0.639	0.590
FOP home	48.91	51.13	48.57	47.81	0.409	0.746
BOP home	50.87	54.08	50.75	51.67	0.581	0.627

**Table 4:** Reading food labels by education.

Reading Labels (%)	Education				ANOVA	
	Primary	Vocational	Secondary	Tertiary	F	*p*
FOP shop	55.89	52.50	55.96	59.08	2.750	**0.042**
BOP shop	51.16	48.21	51.21	52.11	1.209	0.305
FOP home	48.77	48.31	50.38	48.51	0.310	0.818
BOP home	51.72	49.19	54.45	51.15	1.787	0.148

**Table 5:** Reading food labels by professional activity.

Reading Labels (%)	Professional Activity						ANOVA	
	1	2	3	4	5	6	F	*p*
FOP shop	61.16	52.52	48.49	57.98	54.05	55.68	2.725	**0.019**
BOP shop	56.15	48.03	40.80	51.93	49.57	50.58	2.924	**0.013**
FOP home	51.44	48.34	43.73	47.15	47.72	50.73	0.820	0.535
BOP home	55.81	50.34	45.41	53.95	50.55	51.31	1.209	0.303

Notes: 1—white-collar worker, 2—blue-collar worker, 3—unemployed, 4—student, 5—not working and taking care of one’s family, 6—old age pensioner or disability pensioner; the category “other” was excluded from the ANOVA.

**Table 6:** Reading food labels by income.

Reading Labels (%)	Income Intervals						ANOVA	
	1	2	3	4	5	6	F	*p*
FOP shop	48.92	55.33	58.52	55.58	55.01	57.83	2.509	**0.029**
BOP shop	46.66	50.50	50.65	49.49	52.43	53.53	1.034	0.396
FOP home	48.15	49.38	51.31	44.96	50.93	50.15	1.081	0.369
BOP home	47.96	51.54	52.87	52.03	51.56	53.03	0.586	0.711

Notes: the average monthly disposable income of one’s household; 1—below 2000 PLN, 2—2001–3000 PLN, 3—3001–4000 PLN, 4—4001–5000 PLN, 5—5001–6000 PLN, 6—over 6000 PLN.

**Table 7:** Pearson correlation coefficients of reading food labels with the size of one’s household and the number of children.

Number of Persons	FOP Shop		BOP Shop		FOP Home		BOP Home	
	r	*p*	r	*p*	r	*p*	r	*p*
Household	0.076	**0.013**	0.043	0.164	0.066	**0.031**	0.031	0.320
Children	0.043	0.164	0.054	0.081	0.039	0.211	0.040	0.199

Notes: household—the number of people in one’s household, children—the number of children in one’s household.

**Table 8:** Reading food labels by purchasing certain products.

Reading Labels (%)	Yes	No	t	*p*
**Buying dietary supplements**				
FOP shop	57.71	53.11	2.698	**0.007**
BOP shop	51.84	48.85	1.759	0.079
FOP home	51.40	46.86	2.410	**0.016**
BOP home	54.17	49.08	2.716	**0.007**
**Buying organic food**				
FOP shop	58.41	51.73	3.926	**<0.001**
BOP shop	54.71	45.11	5.715	**<0.001**
FOP home	52.92	44.52	4.486	**<0.001**
BOP home	56.05	46.24	5.273	**<0.001**
**Buying functional food**				
FOP shop	56.41	54.75	0.930	0.353
BOP shop	54.15	48.18	3.385	**0.001**
FOP home	52.03	47.43	2.341	**0.019**
BOP home	57.11	48.48	4.434	**<0.001**
**Buying fair trade products**				
FOP shop	57.80	54.41	1.769	0.077
BOP shop	54.31	48.78	2.913	**0.004**
FOP home	50.35	48.57	0.840	0.401
BOP home	56.80	49.56	3.454	**0.001**

Note: the category “No” includes direct “No” answers and “I don’t know” answers.

**Table 9:** Pearson correlation coefficients of reading food labels with the self-rated healthiness of one’s diet, self-rated knowledge about healthy nutrition, self-rated health, and BMI.

Self-Rated Measures	FOP Shop		BOP Shop		FOP Home		BOP Home	
	r	*p*	r	*p*	r	*p*	r	*p*
Diet	0.072	**0.019**	0.104	**0.001**	0.075	**0.014**	0.091	**0.003**
Knowledge	0.089	**0.004**	0.118	**<0.001**	0.087	**0.005**	0.097	**0.002**
Health	0.072	**0.020**	0.088	**0.004**	0.063	**0.042**	0.076	**0.014**
BMI	−0.029	0.344	−0.049	0.109	0.026	0.401	−0.020	0.512

Note: BMI—Body Mass Index (kg/m^2^).

**Table 10:** Reading food labels by being on a special diet for health reasons.

Reading Labels (%)	Yes	No	t	*p*
FOP shop	52.71	55.87	−1.378	0.168
BOP shop	50.83	50.19	0.279	0.780
FOP home	48.55	49.16	−0.242	0.809
BOP home	53.13	51.23	0.753	0.452

**Table 11:** Pearson coefficients of reading food labels with the evaluation of the quantity, understandability, and credibility of selected information on food packaging.

Reading Labels (%)	Quantity		Understandability		Credibility	
	r	*p*	r	*p*	r	*p*
FOP shop	0.033	0.287	0.080	**0.009**	0.083	**0.007**
BOP shop	0.143	**<0.001**	0.079	**0.010**	0.072	**0.019**
FOP home	0.051	0.098	0.073	**0.018**	0.080	**0.010**
BOP home	0.125	**<0.001**	0.080	**0.010**	0.074	**0.017**

**Table 12:** Pearson correlation coefficients of reading food labels with the subjective importance of selected information on labels.

Types of Information	FOP Shop		BOP Shop		FOP Home		BOP Home	
	r	*p*	r	*p*	r	*p*	r	*p*
Health claims	0.078	**0.011**	0.103	**0.001**	0.076	**0.013**	0.102	**0.001**
Nutrition claims	0.081	**0.009**	0.111	**<0.001**	0.088	**0.004**	0.104	**0.001**
List of ingredients	0.091	**0.003**	0.141	**<0.001**	0.084	**0.007**	0.122	**<0.001**
Expiry date	0.093	**0.002**	0.087	**0.005**	0.070	**0.024**	0.081	**0.009**
Country of origin	0.067	**0.029**	0.095	**0.002**	0.080	**0.009**	0.088	**0.004**
Cooking recipes	0.045	0.147	0.053	0.088	0.082	**0.008**	0.059	0.055
Brand	0.070	**0.024**	0.072	**0.020**	0.080	**0.009**	0.074	**0.016**
Organic certificate	0.081	**0.008**	0.102	**0.001**	0.090	**0.003**	0.088	**0.004**
Quality signs	0.084	**0.006**	0.086	**0.005**	0.088	**0.004**	0.094	**0.002**
Recommendations *	0.081	**0.009**	0.092	**0.003**	0.097	**0.002**	0.092	**0.003**
Price	0.075	**0.015**	0.070	**0.023**	0.074	**0.016**	0.078	**0.011**
Average**	0.079	**0.010**	0.095	**0.002**	0.085	**0.006**	0.092	**0.003**

Notes: * of scientific institutes; ** average evaluation of the importance of all types of information mentioned in this column of the table.

**Table 13:** Reading food labels by the most important information on the food label at the first purchase (excluding price).

Reading Labels (%)	Most Important at First Purchase					ANOVA	
	COO	NI	HI	LI	ED	F	*p*
FOP shop	53.52	53.94	54.25	58.68	55.84	1.260	0.284
BOP shop	47.04	53.80	44.34	56.82	47.67	7.257	**<0.001**
FOP home	52.08	47.30	54.56	49.61	48.33	1.145	0.334
BOP home	52.18	54.49	49.47	56.64	47.93	3.878	**0.004**

Notes: COO—the country of origin, NI—nutritional information (e.g., about the content of fat or dietary fiber), HI—information on health effects (e.g., good for the bones, lowering cholesterol), LI—list of ingredients, ED—expiry date; the reading labels indicators were measured in percentages of food products the consumer bought; the responses “other” and “I don’t know” were excluded from the ANOVAs.

**Table 14:** Pearson correlation coefficients of reading food labels with the subjective importance of selected nutritional information on labels.

Types of Information	FOP Shop		BOP Shop		FOP Home		BOP Home	
	r	*p*	r	*p*	r	*p*	r	*p*
Energy value (calories)	0.078	**0.011**	0.120	**<0.001**	0.093	**0.003**	0.111	**<0.001**
Content of fat	0.090	**0.004**	0.128	**<0.001**	0.106	**0.001**	0.130	**<0.001**
Content of sugar	0.088	**0.004**	0.135	**<0.001**	0.097	**0.002**	0.127	**<0.001**
Content of salt	0.071	**0.022**	0.118	**<0.001**	0.104	**0.001**	0.116	**<0.001**
Content of protein	0.095	**0.002**	0.127	**<0.001**	0.100	**0.001**	0.120	**<0.001**
Content of vitamins	0.088	**0.004**	0.123	**<0.001**	0.102	**0.001**	0.123	**<0.001**
Content of dietary fiber	0.096	**0.002**	0.123	**<0.001**	0.106	**0.001**	0.116	**<0.001**
Content of Omega-3 fatty acids	0.082	**0.008**	0.120	**<0.001**	0.104	**0.001**	0.116	**<0.001**

**Table 15:** Pearson correlation coefficients of reading food labels with the subjective importance of selected health information on labels.

Information about the Impact of the Product on:	FOP Shop		BOP Shop		FOP Home		BOP Home	
	r	*p*	r	*p*	r	*p*	r	*p*
Lowering cholesterol	0.071	**0.021**	0.084	**0.007**	0.109	**<0.001**	0.100	**0.001**
Reducing the risk of heart diseases	0.079	**0.010**	0.084	**0.006**	0.100	**0.001**	0.094	**0.002**
Strengthening bones	0.088	**0.004**	0.097	**0.002**	0.099	**0.001**	0.099	**0.001**
The digestive system	0.082	**0.008**	0.101	**0.001**	0.094	**0.002**	0.103	**0.001**
Reducing tiredness and fatigue	0.091	**0.003**	0.097	**0.002**	0.105	**0.001**	0.100	**0.001**
Maintaining proper vision	0.084	**0.007**	0.094	**0.002**	0.104	**0.001**	0.102	**0.001**
Proper development of children	0.075	**0.016**	0.080	**0.009**	0.093	**0.002**	0.085	**0.006**
Proper functioning of the heart	0.080	**0.009**	0.090	**0.004**	0.096	**0.002**	0.100	**0.001**

**Table 16:** Pearson correlation coefficients of reading food labels with the evaluation of the importance of selected promotion instruments for food products with health and nutrition claims.

Promotion Instruments	FOP Shop		BOP Shop		FOP Home		BOP Home	
	r	*p*	r	*p*	r	*p*	r	*p*
TV commercials	0.068	**0.028**	0.055	0.073	0.075	**0.015**	0.056	0.072
Producer website	0.071	**0.020**	0.070	**0.023**	0.088	**0.004**	0.077	**0.012**
Retailer website	0.064	**0.038**	0.064	**0.038**	0.078	**0.012**	0.067	**0.029**
Producer social media profile	0.067	**0.031**	0.055	0.073	0.084	**0.007**	0.067	**0.029**
Retailer social media profile	0.071	**0.022**	0.061	**0.048**	0.086	**0.005**	0.068	**0.028**
Consumer opinions in social media	0.077	**0.012**	0.076	**0.014**	0.083	**0.007**	0.083	**0.007**
Mobile applications	0.059	0.058	0.062	**0.043**	0.080	**0.010**	0.067	**0.031**
Recommendations of family or friends	0.081	**0.009**	0.090	**0.004**	0.080	**0.010**	0.092	**0.003**
Recommendations of a dietician	0.074	**0.016**	0.095	**0.002**	0.073	**0.018**	0.093	**0.002**
Outdoor advertising (e.g., billboards)	0.076	**0.014**	0.063	**0.040**	0.090	**0.004**	0.073	**0.018**
Advertising newsletters of retailers	0.062	**0.045**	0.064	**0.039**	0.063	**0.040**	0.062	**0.043**
Articles in press and magazines	0.068	**0.027**	0.066	**0.032**	0.081	**0.009**	0.069	**0.025**
TV culinary programs	0.085	**0.006**	0.065	**0.034**	0.095	**0.002**	0.072	**0.019**
Culinary blogs	0.075	**0.015**	0.087	**0.005**	0.095	**0.002**	0.089	**0.004**
Product packaging	0.087	**0.005**	0.077	**0.013**	0.078	**0.012**	0.084	**0.007**
Shop assistant	0.076	**0.014**	0.053	0.087	0.077	**0.012**	0.057	0.065

**Table 17:** Pearson correlation coefficients of reading food labels with the subjective importance of selected information types in the marketing communication for food products.

Information Type	FOP Shop		BOP Shop		FOP Home		BOP Home	
	r	*p*	r	*p*	r	*p*	r	*p*
Health effects of consuming a given product	0.099	**0.001**	0.109	**<0.001**	0.101	**0.001**	0.116	**<0.001**
Care for the natural environment	0.087	**0.005**	0.098	**0.001**	0.092	**0.003**	0.088	**0.004**
Supporting producers (e.g., farmers)	0.067	**0.031**	0.073	**0.018**	0.092	**0.003**	0.082	**0.008**
Low price	0.083	**0.007**	0.059	0.056	0.085	**0.006**	0.062	**0.043**
Polish origin of the product	0.057	0.064	0.077	**0.012**	0.088	**0.004**	0.084	**0.007**
Utility of the product in a certain diet	0.087	**0.005**	0.097	**0.002**	0.099	**0.001**	0.085	**0.006**
Above average quality of the product	0.088	**0.004**	0.096	**0.002**	0.083	**0.007**	0.094	**0.002**
Traditional method of production	0.071	**0.021**	0.099	**0.001**	0.089	**0.004**	0.099	**0.001**

**Table 18:** Pearson correlation coefficients of reading food labels with the willingness to pay more for food products with health and nutrition claims compared to conventional products.

WTP	FOP Shop		BOP Shop		FOP Home		BOP Home	
	r	*p*	r	*p*	r	*p*	r	*p*
WTP HC	0.073	**0.018**	0.079	**0.011**	0.077	**0.012**	0.091	**0.003**
WTP NC	0.075	**0.015**	0.087	**0.005**	0.077	**0.012**	0.096	**0.002**

**Table 19:** Selected predictors of reading food labels—full multiple regression models.

Independent Variables	FOP Shop		BOP Shop		FOP Home		BOP Home	
	β	*p*	β	*p*	β	*p*	β	*p*
Woman	−0.004	0.899	0.020	0.525	0.045	0.161	−0.020	0.515
Education	0.031	0.362	−0.070	**0.033**	−0.024	0.490	−0.042	0.205
White-collar	0.050	0.131	0.045	0.149	0.012	0.706	0.029	0.358
Student	0.020	0.556	−0.039	0.222	−0.022	0.521	−0.003	0.924
Income	0.041	0.208	0.042	0.175	0.024	0.462	0.026	0.405
Household	−0.009	0.776	−0.032	0.306	−0.041	0.218	−0.054	0.092
Dietary suppl.	0.035	0.259	−0.001	0.961	0.021	0.507	0.027	0.365
Organic	0.036	0.298	0.039	0.237	0.065	0.060	0.011	0.747
Functional	−0.034	0.304	0.018	0.562	0.012	0.712	0.042	0.193
Fair trade	0.010	0.763	0.032	0.314	−0.025	0.453	0.043	0.183
Diet	−0.041	0.292	0.035	0.347	−0.029	0.454	0.030	0.420
Knowledge	0.117	**0.002**	0.131	**<0.001**	0.110	**0.004**	0.062	0.094
Health	−0.002	0.955	0.060	0.067	−0.005	0.879	0.044	0.180
Quantity	0.025	0.459	0.067	**0.037**	0.021	0.523	0.069	**0.034**
Understandability	0.042	0.253	0.026	0.452	0.011	0.760	0.054	0.127
Credibility	0.056	0.144	−0.063	0.088	0.037	0.336	−0.033	0.380
List of ingredients	−0.022	0.565	0.170	**<0.001**	−0.033	0.400	0.103	**0.006**
Expiry date	0.112	**0.001**	−0.002	0.956	0.003	0.941	0.012	0.727
Institutes	0.017	0.643	−0.014	0.690	0.053	0.147	−0.005	0.890
First-time LI	0.048	0.124	0.085	**0.004**	−0.006	0.852	0.060	**0.048**
Sugar	−0.049	0.318	0.056	0.228	−0.037	0.455	0.045	0.344
Fat	0.038	0.466	0.055	0.266	0.091	0.079	0.108	**0.031**
Dietary fiber	0.043	0.338	0.016	0.701	0.033	0.462	−0.031	0.481
Cholesterol	−0.045	0.325	−0.071	0.098	0.077	0.089	0.010	0.813
Digestive system	−0.070	0.172	0.008	0.865	−0.108	**0.037**	−0.027	0.590
Fatigue	0.070	0.128	0.018	0.685	0.091	**0.046**	−0.001	0.988
Dietician	−0.058	0.130	0.003	0.933	−0.107	**0.005**	−0.018	0.628
Blogs	0.028	0.461	0.033	0.364	0.099	**0.010**	0.039	0.293
Packaging	0.055	0.116	−0.035	0.296	0.020	0.574	0.008	0.814
Health effects	0.110	**0.009**	0.055	0.166	0.094	**0.025**	0.104	**0.010**
WTP HC	−0.004	0.941	−0.029	0.594	−0.011	0.847	−0.007	0.900
WTP NC	−0.027	0.640	−0.006	0.907	−0.071	0.214	0.000	0.994
R^2^	0.091		0.173		0.089		0.143	

**Table 20:** Selected predictors of reading food labels—retrograde step regression models.

Independent Variables	FOP Shop		BOP Shop		FOP Home		BOP Home	
	β	*p*	β	*p*	β	*p*	β	*p*
White-collar	0.072	**0.017**	x	x	x	x	x	x
Functional	x	x	x	x	x	x	0.062	**0.039**
Knowledge	0.115	**<0.001**	0.168	**0.031**	0.105	**0.031**	0.107	**0.001**
Health	x	x	0.065	**0.030**	x	x	x	x
Quantity	x	x	0.076	**0.029**	x	x	0.062	**0.037**
Credibility	0.066	**0.040**	x	x	x	x	x	x
List of ingredients	x	x	0.175	**0.033**	x	x	0.120	**<0.001**
Expiry date	0.116	**<0.001**	x	x	x	x	x	x
First-time LI	x	x	0.086	**0.029**	x	x	x	x
Fat	x	x	0.094	**0.033**	0.081	**0.036**	0.113	**0.001**
Cholesterol	x	x	x	x	0.086	**0.036**	x	x
Dietician	x	x	x	x	−0.092	**0.037**	x	x
Blogs	x	x	x	x	0.107	**0.035**	x	x
Health effects	0.096	**0.003**	x	x	0.081	**0.037**	0.106	**0.002**
R^2^	0.066		0.152		0.068		0.124	

Note: only predictors significant at ***p* < 0.05** are included in these models.

## Appendix A

**Table A1:** A comparison of the sample with the general population (%).

Characteristics	Categories	General Population	Study Sample
Sex	Women	52.1	53.3
	Men	47.9	46.7
Age	15–24	12.5	15
	25–34	17.8	17.3
	35–44	18.7	17.9
	45–54	14.5	13.9
	55–64	16.5	15.7
	65 and more	20	20.2
Education	Primary	49.4	47.7
	Secondary	31.9	31.6
	Tertiary	18.7	20.7
Place of living	Urban areas	60.7	61.7
	Rural areas	39.3	38.3
Region	Dolnośląskie	7.6	7.6
	Kujawsko-pomorskie	5.4	5.6
	Lubelskie	5.6	5.6
	Lubuskie	2.6	2.7
	Łódzkie	6.5	6.7
	Małopolskie	8.7	8.6
	Mazowieckie	13.9	13.5
	Opolskie	2.6	2.8
	Podkarpackie	5.5	5.6
	Podlaskie	3.2	3.3
	Pomorskie	5.9	6
	Śląskie	11.9	11.4
	Świętokrzyskie	3.4	3.4
	Warmińsko-mazurskie	3.7	3.8
	Wielkopolskie	9	9
	Zachodniopomorskie	4.5	4.4

Note: general population refers to the inhabitants of Poland aged 15 and more.

**Table A2:** The operationalization of key variables used in this study.

Variable	Operationalization	Measurement Scale and Coding
FOP shop	While shopping, you read the information on the front of the packaging for what share of food products you buy?	%
BOP shop	While shopping, you read the information on the back of the packaging for what share of food products you buy?	%
FOP home	At home, you read the information on the front of the packaging for what share of food products you have bought?	%
BOP home	At home, you read the information on the back of the packaging for what share of food products you have bought?	%
Woman	Sex	Woman—1, Man - 0
Education	Education level	Primary—1, Vocational—2, Secondary—3, Tertiary—4
White-collar	Professional activity—a single-choice question with the following catalog of answers: (a) white-collar worker, (b) blue-collar worker, (c) unemployed, (d) student, (e) I don’t work and I take care of the family, (f) old age pensioner/disability pensioner, (g) other. What?	White-collar worker selected—1, White-collar worker not selected—0
Student	Professional activity—a single-choice question with the following catalog of answers: (a) white-collar worker, (b) blue-collar worker, (c) unemployed, (d) student, (e) I don’t work and I take care of the family, (f) old age pensioner/disability pensioner, (g) other. What?	Student selected—1, Student not selected—0
Income	What is the total monthly net, i.e., disposable, income of all members of your household? A single-choice question with the following catalogue of answers: (a) below 2000 PLN, (b) 2001–3000 PLN, (c) 3001–4000 PLN, (d) 4001–5000 PLN, (e) 5001–6000 PLN, (f) over 6000 PLN	(a) below 2000 PLN—1, (b) 2001–3000 PLN—2, (c) 3001–4000 PLN—3, (d) 4001–5000 PLN—4, (e) 5001–6000 PLN—5, (f) over 6000 PLN—6
Household	Size of your household	Number of persons
Dietary suppl.	Do you buy the following products? Dietary supplements	Yes—1, No—0, I don’t know—0
Organic	Do you buy the following products? Organic food	Yes—1, No—0, I don’t know—0
Functional	Do you buy the following products? Functional food	Yes—1, No—0, I don’t know—0
Fair trade	Do you buy the following products? Fair trade products	Yes—1, No—0, I don’t know—0
Diet	How do you evaluate your diet?	Very healthy—5, Rather healthy—4, Average—3, Rather unhealthy—2, Very unhealthy—1
Knowledge	How do you evaluate your knowledge about healthy nutrition?	Very big—5, Rather big—4, Average—3, Rather small—2, Very small—1
Health	How do you evaluate your health status?	Very good—5, rather good—4, average—3, rather poor—2, very poor—1
Quantity	How do you assess the quantity of the following information on food product packages?	Measured separately for health claims, nutrition claims, and quality signs Too much—1, Appropriate—2, Too little—3 The arithmetical mean
Understandability	How understandable for you is the following information on food product packages?	Measured separately for health claims, nutrition claims, quality signs, functional food, and organic food Very understandable—5, Rather understandable—4, Average—3, Rather not understandable—2, Completely not understandable—1 The arithmetical mean
Credibility	How credible for you is the following information on food product packaging?	Measured separately for health claims, nutrition claims, list of ingredients, expiry date, organic certificate, and quality signs Very credible—5, Rather credible—4, Average—3, Rather not credible—2, Definitely not credible—1 The arithmetical mean
List of ingredients	How important for you is the following information on food product packages? List of ingredients	Very important—5, Rather important—4, Average—3, Rather not important—2, Without any importance—1
Expiry date	How important for you is the following information on food product packages? Expiry date	Very important—5, Rather important—4, Average—3, Rather not important—2, Without any importance—1
Institutes	How important for you is the following information on food product packages? Recommendations of scientific institutes	Very important—5, Rather important—4, Average—3, Rather not important—2, Without any importance—1
First-time LI	What constitutes the most important information on a food product label when you buy it for the first time (excluding price)? A single-choice question with the following answer options: (a) country of origin, (b) nutritional information (e.g., about the content of fat or dietary fiber), (c) information about health effects (e.g., good for the bones, lowering cholesterol), (d) list of ingredients, (e) expiry date, (f) other, (g) I don’t know	List of ingredients selected—1, List of ingredients not selected—0
Sugar	How important for you is the following information on food product packages? Content of sugars	Very important—5, Rather important—4, Average—3, Rather not important—2, Without any importance—1
Fat	How important for you is the following information on food product packages? Content of fats	Very important—5, Rather important—4, Average—3, Rather not important—2, Without any importance—1
Dietary fiber	How important for you is the following information on food product packages? Dietary fiber	Very important—5, Rather important—4, Average—3, Rather not important—2, Without any importance—1
Cholesterol	How important for you is the following information on food product packages? Impact of the product on lowering cholesterol	Very important—5, Rather important—4, Average—3, Rather not important—2, Without any importance—1
Digestive system	How important for you is the following information on food product packages? Impact of the product on the digestive system	Very important—5, Rather important—4, Average—3, Rather not important—2, Without any importance—1
Fatigue	How important for you is the following information on food product packages? Impact of the product reducing tiredness and fatigue	Very important—5, Rather important—4, Average—3, Rather not important—2, Without any importance—1
Dietician	In your opinion, how important are the following ways of promoting food products with health and nutrition claims? Recommendations of a dietician	Very important—5, Rather important—4, Average—3, Rather not important—2, Without any importance—1
Blogs	In your opinion, how important are the following ways of promoting food products with health and nutrition claims? Culinary blogs	Very important—5, Rather important—4, Average—3, Rather not important—2, Without any importance—1
Packaging	In your opinion, how important are the following ways of promoting food products with health and nutrition claims? Food packages	Very important—5, Rather important—4, Average—3, Rather not important—2, Without any importance—1
Health effects	How important for you is the following information concerning food products? Health effects of consuming a given product	Very important—5, Rather important—4, Average—3, Rather not important—2, Without any importance—1
WTP HC	Are you willing to pay more for products with health claims (compared to similar products without such claims)?	Definitely yes—5, Rather yes—4, I don’t know—3, Rather not—2, Definitely not—1
WTP NC	Are you willing to pay more for products with nutrition claims (compared to similar products without such claims)?	Definitely yes—5, Rather yes—4, I don’t know—3, Rather not—2, Definitely not—1
